# Combining Immune Checkpoint Inhibitors with Anti-Angiogenic Agents

**DOI:** 10.3390/jcm9030675

**Published:** 2020-03-03

**Authors:** Paola Ciciola, Priscilla Cascetta, Cataldo Bianco, Luigi Formisano, Roberto Bianco

**Affiliations:** 1 Department of Clinical Medicine and Surgery, University of Naples “Federico II”, 80131 Naples, Italy; paola.ciciola@gmail.com (P.C.); priscillacascetta@gmail.com (P.C.); 2 Department of Experimental and Clinical Medicine, University of Catanzaro “Magna Graecia”, 88100 Catanzaro, Italy; bianco@unicz.it

**Keywords:** ICIs, angiogenesis, immunotherapy

## Abstract

Immunotherapy has recently emerged as a novel strategy for treating different types of solid tumors, with promising results. However, still a large fraction of patients do not primarily respond to such approaches, and even responders sooner or later develop resistance. Moreover, immunotherapy is a promising strategy for certain malignancies but not for others, with this discrepancy having been attributed to a more immunogenic microenvironment of some tumors. As abnormal and augmented tumor vessels often occur in cancerogenesis, anti-angiogenic drugs have already demonstrated their effectiveness both in preclinical and in clinical settings. By targeting abnormal formation of tumor vessels, anti-angiogenetic agents potentially result in an enhanced infiltration of immune effector cells. Moreover, crosstalks downstream of the immune checkpoint axis and vascular endothelial growth factor receptor (VEGFR) signaling may result in synergistic effects of combined treatment in tumor cells. In this review, we will describe and discuss the biological rationale of a combined therapy, underlying the modification in tumor microenvironment as well as in tumor cells after exposure to checkpoint inhibitors and anti-angiogenic drugs. Moreover, we will highlight this strategy as a possible way for overcoming drug resistance. By first discussing potential prognostic and predictive factors for combined treatment, we will then turn to clinical settings, focusing on clinical trials where this strategy is currently being investigated.

## 1. Introduction

### 1.1. Immune Checkpoint Inhibitors

Increasing the activation of T cell effectors is essential in the immune response to cancer. T cell activation is a multistep process that is triggered by the initial recognition of antigenic peptide–MHC complexes by the T cell receptor, followed by the delivery of secondary costimulatory signals to fully activate the T cell [1,2]. T cell activation can also be inhibited by negative regulatory molecules, also referred to as checkpoint molecules, which can override primary and secondary T cell activation signals [1]. Multiple T cell checkpoint molecules have been described, and the blockade of either of of these two inhibitory proteins, cytotoxic T-lymphocyte antigen 4 (CTLA4) and programmed cell death protein 1 (PD-1), has resulted in clinical benefit in several tumor types [3,4,5,6].

CTLA4 (CD152) is a membrane glycoprotein expressed by immunosuppressive T regulatory cells (Tregs) that inhibits early T cell activation and has an important role in the priming phase of the immune response [7,8]. In preclinical studies, the blockade of CTLA4 led to a 1.5- to two-fold increase in the proliferation of T cells, a six-fold increase in the production of interleukin-2 [9,10] and the depletion of T regulatory lymphocytes in the tumor microenvironment through a macrophage-dependent process [10,11]. Ipilimumab, an antibody that inhibits CTLA4 interactions with its ligands CD80 and CD86, is approved for the treatment of patients with advanced melanoma, based on clinical studies that demonstrated improvements in overall survival in a subset of patients [12,13,14]. PD1 (CD279) is an inhibitory co-receptor expressed on the cell surface of T lymphocytes CD8+ and CD4+, natural killer cells (NK), B lymphocytes, and tumor-infiltrating lymphocytes (TILs) [15].

PD-1 plays a key role in balancing tumor immunity and inflammatory reactions, in fact it blocks T cell activation and is associated with chronically activated and exhausted T cells, such as those found in the tumor microenvironment [16,17,18]. PD-1 interacts with two ligands: PD-L1 (CD274), expressed on the cell surface of activated lymphocytes (T, B, and NK) [19], peripheral tissues and organs [20], and to a greater extent by tumor cells, and PD-L2, expressed primarily by macrophages and dendritic cells [21]. The expression of PD-1 by exhausted T cells indicates their lost capability to execute their effector function, while the interaction between PD-1 and PD-L1/2 leads to the inhibition of T cell activation and cytokine secretion, i.e., interferon-γ (IFN-γ), tumor necrosis factor-α (TNF-α), and interleukin 2 (IL-2), and helps to maintain immune homeostasis by avoiding the onset of autoimmunity [22].

Multiple antibodies that inhibit PD-1 or PD-L1 are in clinical development [23,24]. Nivolumab and pembrolizumab, two antibodies that target PD-1 and block its interactions with PD-L1 and PD-L2, are approved for the treatment of advanced melanoma, non-small cell lung cancer (NSCLC), and renal cell carcinoma (RCC), based on clinical studies that demonstrated improvements in overall survival [25,26]. The exact determinants of response to anti-PD1 and anti-PDL1 therapies are not well understood. However, clinical benefit is associated with high tumor mutational load [27], high pretreatment levels of PD-L1 on tumor cells, and tumor-infiltrating immune cells [28,29,30], and high pretreatment levels of tumor-infiltrating lymphocytes [31].

Although the results from some studies about the association of PD-L1 expression and the prognosis for several tumor type have been demonstrated to be not very conclusive, blocking the interaction between PD-1 and PD-L1 may induce to favorable effect on tumor treatment by the reactivation of cytotoxic T lymphocytes and the restoration of their ability to attack cancer cells. So focusing on the effect of the immune checkpoint inhibitors on tumor progression is emerging.

### 1.2. Anti-Angiogenic Agents

Angiogenesis is an essential process for the proliferation of solid tumors [32]. Pre-clinical studies showed that tumors induce the formation of sprouting vessels from the surrounding vasculature and that this process is vital for the growth of tumors beyond 2–3 mm^3^ in size [33]. Several molecules have been identified as angiogenic factors. In particular, the isolation and the cloning of endothelial growth factor-A (VEGF) [34,35] led to great progress to understanding angiogenic mechanisms that sustain tumor growth. VEGFA is a growth/survival factor for endothelial cells and binds to two receptor tyrosine kinases (RTKs), VEGF receptor (VEGFR) 1 and 2 [36]. VEGFR2 is expressed on endothelial cells whereas VEGFR1 is expressed on endothelial cells and other cell types, such as smooth muscle cells, fibroblasts, myeloid progenitors, macrophages, and various types of cancer cells [37].

Several animal studies have shown that VEGF factors are overexpressed in most solid cancers and that the inhibition of the VEGF signaling pathway can suppress tumor growth [38].

Based on these observations, numerous therapies that target angiogenesis have been developed, mainly by blocking the VEGF signaling pathway. In 1993, a monoclonal neutralizing antibody against VEGFA was reported to inhibit tumor growth in a xenograft model [39]. This idea led to the development of bevacizumab (Avastin), a recombinant humanized monoclonal antibody specific to VEGFA. In 2004, bevacizumab was approved by the U.S. Food and Drug Administration (FDA) for the treatment of metastatic colorectal cancer (CRC) [40]. In addition, various other inhibitors of the VEGF signaling pathway have been developed. The RTK inhibitors (RTKIs) sunitinib (Sutent) [41], sorafenib (Nexavar) [42], and pazopanib (Votrient) [43] are currently approved for the treatment of various types of cancers. Ramucirumab (Cyramza) is also a monoclonal antibody that binds VEGFR2 to block the VEGF signaling pathway and has been approved by the FDA for the treatment of several types of solid cancers [44].

Despite a large amount of promising data from animal experiments, simply blocking the VEGF signaling pathway by an anti-VEGF/R monotherapy appears to be ineffective for advanced cases in the clinical setting [45].

Primary or de novo treatment resistance has often involved in the treatment of cancer patients, even with the most recent sophisticated drugs. Mechanisms related to the resistance to anti-VEGF/R therapy are not completely unveiled. However, it is known that in anti-angiogenic therapy resistance often involves the activation of signaling pathways other than the VEGF pathway. Intra-tumor hypoxia and the related infiltration of immunosuppressive cells seem to be largely responsible for the angiogenic relapse and drug resistance [46,47]. In this context, novel therapeutic approaches aimed to target the tumor vasculature and to overcome anti-angiogenic therapy resistance have been explored [48,49].

Particularly, immune checkpoint inhibitors have been designed to target tumor microenvironment but they need to be clinically validated as treatment strategies for anti-VEGF/R therapy resistant tumors. Taken together, many studies suggest that several solid tumors, especially advanced stage non-small cell lung cancer (NSCLC) [50] and renal cell cancer (RCC) [51], are responsive to both anti-VEGF and anti-PD-1/PD-L1 therapies. Therefore, it is of high interest to further explore the therapeutic effect of combining anti-angiogenic drugs with immunotherapeutic agents.

## 2. Biological Rationale of Combined Therapy

Earlier studies have suggested that anti-angiogenic therapy can elicit or enhance tumor immunity response, whereas reciprocally the immune system can support angiogenesis [6,52,53]. So, combining anti-angiogenic agents and immunotherapy could be synergistic. [52].

### 2.1. Crosstalk between Angiogenesis and Immune System

Immune activity in tumors is mainly regulated by PD-1/PD-L1 interactions [54] and it is well known that the upregulation of this immune checkpoint signaling pathway protects cancer from immune surveillance [55]. In addition, pro-angiogenic factors can play a direct and indirect role in immunosuppressive tumor microenvironment [54].

Various pro-angiogenic molecules have been shown to be associated with a range of immunosuppressive effects at successive steps in the cancer-immunity cycle, such as antigen presentation, T cell priming, T cell trafficking, and T cell tumor infiltration. The molecules that regulate angiogenesis can affect immune cells and their interaction with tumors in at least three ways: (a) direct effects when they bind their cognate receptors expressed by immune cells; (b) indirect effects when they induce changes in protein expression on endothelial cells; and (c) indirect physical effects through the promotion of vascular normalization or the reduction of neoangiogenesis [56].

Regarding the direct effects of VEGF on immune cells, it has been described that VEGF can inhibit dendritic cell maturation inducing potential immune evasion by tumors [57]. In vitro studies have shown that treatment with the anti-VEGF monoclonal antibody bevacizumab or the multityrosine-kinase inhibitor (TKI) of VEGFR2 sorafenib can restore the differentiation of monocytes into dendritic cells through VEGF expression regulation [58]. VEGF can also directly upregulate PD-L1 expression on dendritic cells leading to the reduction of the function and/or the number of T cells [59]. In preclinical studies, VEGF has been reported to have direct effects on T cell function, such as the inhibition of the differentiation of hematopoietic progenitor cells in the thymus into CD8+ and CD4+ T cells [60]. Moreover, VEGF binding to VEGFR2 on the surface of effector T cells has been shown to directly suppress their proliferation and cytotoxic function by the upregulation of the expression of PD-1 on CD8+ T lymphocytes [61]. The last direct effect described is related to VEGF binding to VEGFR2 on Treg cells and on myeloid-derived suppressor cells (MDSCs). This binding can support the infiltration of these immunosuppressive cells in tumor microenvironment [62]. VEGF can also bind to the VEGF co-receptor neuropilin1, which is expressed by Treg cells, and this interaction is critical for tumor homing [63].

Morphologically, tumor blood vessels are tortuous, dilated, and unevenly distributed, with adjacent endothelial cells being loosely attached to one another. Pericytes, which surround the blood vessels and regulate vascular permeability, are usually detached from the endothelial cells, resulting in leaky tumor blood vessels that are characterized by dysfunctional flow characteristics [64,65]. In tumor-associated endothelial cell VEGF can modulate adhesion molecules expression, such as vascular cell adhesion molecule-1 (VCAM1) and intercellular adhesion molecule-1 (ICAM1), and chemokines expression, ultimately promotes the creation of a specific barrier that serves as an impermeable block to certain immune cells [66,67,68]. The creation of a selective immunosuppressive barrier depends also by the expression of immune-checkpoint molecules, such as PD-L1 and PD-L2, that may be upregulated in tumor endothelial cells [69].

It has been extensively described that the upregulation of VEGF expression in tumor cells promotes neovascularization and the growth and metastasis of solid tumors [70]. Due to the rapid division and growth, tumor cell consume a large amount of oxygen and nutrients with consequent hypoxia and acidosis in the tumor bed [54,70]. This contributes to the immunosuppressive effect of the tumor microenvironment, and in vitro and in vivo mouse models, hypoxia leads to PD-L1 upregulation [71], enhances the activity of suppressor T regulatory cells and inhibits effector T cell functions [72]. On the other hand, several studies suggest that the immune system can exert many effects on tumor angiogenesis [73,74,75]. The environment surrounding tumor cells is characterized by the chronic overexpression of inflammatory mediators, and the immune system struggles to recognize aberrant cells and remove them, i.e., immune cells become unresponsive to tumor cells [76]. Considering the role of immune system in cancer, several routes could be used to tackle tumor progression: (a) inhibition of macrophage recruitment into tumor tissues; (b) inhibition of macrophage differentiation into the pro-tumoral phenotype (tumor-associated macrophages, TAMs); and (c) targeting chronic inflammation or pro-tumorigenic factors supplied by adaptive immune cells [77].

Monocytes are recruited to tumors by both malignant stromal or tumor cell-derived chemokines and growth factors and can differentiate in macrophages. Several studies have shown that tumors were markedly less vascularized when their murine hosts were depleted of monocytes [78,79,80]. Moreover, it has been demonstrated that macrophages can produce many factors with mitogenic effects on endothelial cells such as fibroblast growth factor (FGF), VEGF, and matrix metallopeptidase-9 (MMP-9), a metalloprotease expressed by infiltrating macrophages that is implicated in the release and mobilization of VEGF from extracellular matrix [81].

Tumor angiogenesis can be influenced also by secretion of pro-inflammatory cytokines. Much evidence has suggested that IL-1, IL-6, and IL-17 induce angiogenesis indirectly through increases of VEGF expression [82] or through the activation of signal transducer and activator of transcription 3 (STAT3), a transcription factor expressed by tumor cells which can regulate the production of VEGF [83,84].

Finally, MDSCs are important players in the immune response against tumors [85]. In fact, the elevated release of VEGF by cancer cells induce the production of MDSCs in the bone marrow that are kept undifferentiated in the tumor microenvironment (TME) [86]. The presence of MDSCs in the TME is usually associated with poor prognosis [87]. MDSCs are precursors of dendritic cells, macrophages, and granulocytes, and they can differentiate into pro-tumoral phenotype under hypoxic conditions [87,88].

All these finding suggest that immune-checkpoint blockade combined with anti-angiogenic therapy might induce the normalization of blood vessels and also improve immune cell response to tumor progression.

### 2.2. Modifications in Tumor Microenvironment after Exposure to Combined Therapy

**Figure 1:**
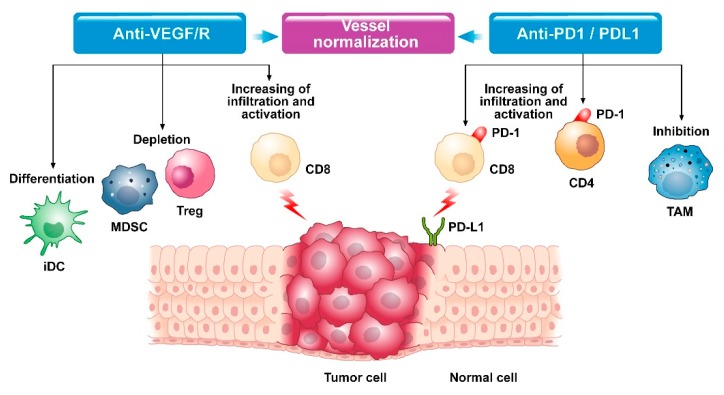
Modifications in the tumor microenvironment after combined anti-VEGF and anti-PD1/PDL1 therapy. iDC = immature dendritic cell; MDSC = myeloid-derived suppressor cell; Treg = regulatory T cell; CD8 = linfociti T CD8; CD4 = linfociti T CD4; TAM = Tumor-associated macrophage; PD-1 = programmed cell death protein 1; PD-L1 Programmed death-ligand 1.

The aberrant tumor vasculature is a major and consistent hallmark of solid tumors. It is well recognized that the most widely used approach for vascular normalization is the blockade of VEGF or its receptors via anti-angiogenic agents [65]. In preclinical setting the resulting normalized vessel function increases pericyte coverage and enhances tumor perfusion, thus supporting more homogeneous oxygen and immune cells delivery [65,89]. Vascular normalization can convert the immunosuppressive TME into an immune-stimulatory one by promoting the accumulation, penetration, and antitumor activity of immune effector cells, and by reducing hypoxia and function of suppressive cells (Figure 1) [54].

Combining immunotherapy of anti-PD-L1 with anti-angiogenic therapy had reciprocal beneficial effects: anti-angiogenic drugs block the negative immune signals by increasing ratio of anti-/pro-tumor immune cells and decreasing immune checkpoints expression, while immunotherapy restores immune-supportive microenvironment and promotes vascular normalization increasing lymphocyte infiltration and activation [64].

Several studies have shown that treatment of xenograft cancer models with inhibitors of VEGF-A or VEGFRs increases T cell recruitment and infiltration into tumors [90,91,92] and can exert a synergistic antitumor effect with anti-PD1 therapy [93].

Treatment with a dual anti-CTLA4 and anti-PD1 blockade induces tumor vessel normalization. Tian and colleagues reported that the antitumor effects of immune checkpoint blockade are related to their influence on T cells but they could also derive from their ability to remodel tumor vasculature in breast cancer [94]. Checkpoint inhibitors seem to lead to vascular normalization via the induction of type 1 T-helper (TH-1) cells in the TME, which have been shown to co-localize with tumor endothelial cells and induce changes in the cytokine environment, and subsequently affect pericyte recruitment/attachment. Furthermore, vessel normalization leads to changes in the immune cell composition governed by recruiting T lymphocytes and decreasing neutrophils [94]. Recently, Allen and coworkers treated refractory pancreatic, breast and brain tumor mouse models with combined therapy using PD-1/PD-L1 pathway blockers and anti-angiogenic agents, since an increased expression of PD-L1 was observed after anti-angiogenic treatment. Interestingly, they found that anti-PD1 therapy sensitized and prolonged the efficacy of the anti-angiogenic therapy in pancreatic and breast cancer models. On the other hand, the anti-angiogenic therapy improved anti-PD-L1 treatment, especially by the increased cytotoxic T cell infiltration due to the formation of intra-tumoral high endothelial venules induced by the therapy [95].

Several pieces of evidence suggest that anti-angiogenic therapies can reverse endothelial cell anergy and induce adhesion molecule expression and immune infiltration, highlighting the complex interplay between tumor angiogenesis and anti-tumor immunity [96].

The polarization of the immunosuppressive TME into an immune-supportive environment needs also the depletion of Tregs in order to activate CD8+ T cells. VEGF-A/VEGFR2 targeted therapies can modulate immunosuppressive cells (i.e., Treg and MDSC): they can enhance the proportion of tumor-infiltrating T lymphocytes probably by normalizing tumor vessels and by modulating the expression of adhesion molecules involved in T-lymphocyte extravasation [97]. In line with these observations, Ozao-Choy and colleagues have shown that sunitinib, a VEGFR2 inhibitor, is able to modulate the tumor microenvironment not only by decreasing Treg and MDSC levels inside the tumor but also by down-regulating cytokines, such as IL-10 and TGF-β, and the immune suppressive costimulatory receptors, such as PD-1 and CTLA4 [98].

Overall, in preclinical models the normalization of tumor vasculature with anti-VEGF antibody can increase extravasation of adoptively transferred T cells into the tumor and to improve the clinical efficacy of adoptive cell transfer-based immunotherapy [92].

Many others studies have focused on exploring these potential combinatorial strategies with synergistic antitumor activity [51,99,100,101]. For example, in an in vivo lung adenocarcinoma model, immunotherapy combined with bevacizumab synergistically inhibits tumor growth [100]. Anti-PD-L1 mAb combined with VEGFR2 small molecule inhibitor can significantly downregulate the expression of PD-1 and PD-L1, increase tumor-infiltrating lymphocytes, and inhibit tumor growth by reducing Tregs and MDSCs [102]. Moreover, Merder and colleagues conducted a preclinical study in genetically engineered small-cell lung cancer (SCLC) mouse models: combination therapy group exerted the best survival outcome. Compared with mice sensitive to anti-PD-L1 treatment, the abundance of exhausted T cell (PD-1+/TIM-3+/LAG-3+ T cell) significantly increased in mice resistant to anti-PD-L1. However, increased ratio of exhausted T cells was counteracted by anti-VEGF plus anti-PD-L1 treatment [103].

As for renal cancer, it has been shown that the TKI sunitinib reverses type-1 immune suppression and decreases Treg in RCC patients [104]. In a recent study it has been demonstrated that anti-PDL1 atezolizumab in combination with bevacizumab enhances antigen-specific T-cell migration in metastatic renal cell carcinoma [105].

Lastly, the use of anti-PD-1 antibodies in combination with anti-VEGFR antibodies in the CT26 mouse model of CRC resulted in improved antitumor effects, with an average of an approximate 75% reduction in tumor growth compared with control treatment. Similarly, in mice with tumors derived from injection of mouse colon cancer C26 cells, treatment with a combination of anti-VEGFR2 and anti-PD-1 monoclonal antibodies led to enhanced inhibition of tumor growth compared with either treatment alone [93].

Overall, to date, the biological background of the complex and dynamic interactions of targeting tumor microenvironment and inducing anti-tumor immune response have not been sufficiently investigated [106].

### 2.3. Combined Therapy as a Way to Overcome Cell Resistances

As discussed above several anti-angiogenesis drugs have been approved for treatment of several solid tumors [40,41,42,43,44]. Although these VEGF pathway inhibitors can improve survival in most cancer patients, some of them have little or no beneficial effect from such therapies. It has been described that host immune cells such as tumor-associated macrophages contribute to some mechanisms of anti-VEGF therapy resistance [107,108,109].

Anti-angiogenesis therapies stimulate Teff cells infiltration but this effect may be blunted by concomitant recruitment of immunosuppressive immune cells and by up-regulation, on tumor cell surface, of PDL-1 which in turn inhibits Teff activity [110]. Increased PDL-1 expression has been observed in anti-angiogenetics treated tumors such as sunitinib-treated RCC cell lines and xenografts [110]. These findings suggest that immune system could be involved and could promote resistance to anti-angiogenic agents. Therefore, strategies targeting the immunosuppressive PD-1/PDL-1 signaling in anti-angiogenesis resistant tumors, aimed to relieve Teff cell suppression, are emerging.

Recent studies suggest that the dual blockade of angiopoietin-2 and VEGF increases PDL1 expression in tumor endothelial cells [111]. This observation raises the possibility that resistance to anti-angiogenic therapy may arise, at least in part, from the development of adaptive immune suppressive processes within tumors [95,111] and that antitumor efficacy of a simultaneous blockade could be further enhanced by anti-PD-1 treatment, in different tumor models [111].

Metastatic RCCs often develop resistance to the use of tyrosine kinase inhibitors (TKIs), so immune checkpoint blockade is becoming a point of interest [48]. The rationale is to restore the patient’s natural tumor-specific T cell–mediated immune responses by neutralizing any inhibitory signaling [48].

Nivolumab, a PD-1 monoclonal antibody approved for patients with metastatic melanoma and lung cancers, has also been approved in the treatment of metastatic RCC [112,113]. Nivolumab neutralizes the interaction between PD-1 and its ligands PD-L1 and PD-L2 [112], normally responsible for the downregulation of cellular immune response [51]. Sunitinib-nivolumab and pazopanib-nivolumab combinations have been tested in patients with advanced metastatic RCC [49]. Similarly, pre-treatment versus post-treatment samples from patients with RCC treated with sunitinib showed decreases in Tregs after each treatment cycle confirming the role of TKIs in immunomodulation of the tumor microenvironment [114,115].

### 2.4. A Predictive Value of PD-L1 Expression

Clinically, PDL1 expression is associated with poorer prognosis in a variety of solid tumors, such as melanoma, renal cancer, and lung cancer [116,117]. The degree of PD-L1 expression on the tumor and immune cells in the microenvironment may reflect dependence of tumor on this pathway. As such, PD-L1 expression on tumor cells, tumor-infiltrating cells, and surrounding microenvironment immune cells is currently being evaluated also as a predictor of response. However, conclusions from some trails focusing on PD-L1 expression as predictor factor are inconsistent and conflicting. Some studies have found high relation between PD-L1 expression and increased response rate and clinical benefit from anti-PD-1/anti-PD-L1 therapy [118,119]. In contrast, Motzer and colleagues have observed no correlation between PD-L1 expression and survival benefit in patients receiving nivolumab plus ipilimumab in the CheckMate 214 trial, where a longer PFS rate was observed in patients receiving immunotherapy with PD-L1 expression of 1% or more [120]. PD-L1 expression (positive/negative) is measured by proportion of PD-L1 expressing tumor cell and/or immune cell [118,119]. Due to intra-tumoral heterogeneity and dynamic alteration of PD-L1 expression, cancer progression, and modifications induced by treatments, the actual status of PD-L1 could be misinterpreted [121,122]. Heterogeneous distribution of PD-L1 expressing tumor or stromal cell results in discordance between biopsy specimen and resection tissue [123]. Therefore, when resection tissue is not available, PD-L1 expression of the whole tumor microenvironment might be displayed inaccurately [123,124].

Expression of PD-L1 variates during cancer evolution and treatment and it is generally believed as a surrogate of pre-existing immune specific immune activity and can be upregulated by IFN-γ in tumor microenvironment. Other factors simultaneously can influence PD-L1 expression such as intracellular oncogenic signaling pathway apart from adaptive immune resistance [125].

Multiple studies have evaluated PD-L1 predictive capacity in patients treated with VEGF-targeted therapies. In particular, PD-L1 expression has been evaluated as a predictor of response in 453 patients with metastatic ccRCC undergoing treatment with sunitinib or pazopanib in the first-line setting in a phase 3 trial [126]. PD-L1 expression was quantified in the tumor cells and in TAMs using a somewhat complicated H-score. Any degree of PD-L1 expression was seen in 36% of patients and was associated with increased infiltration with TAMs, compared with lower expression. Increased PD-L1 expression (H-score > 50) was associated with shorter overall survival (pazopanib high/low: 32 months versus 20 months; sunitinib high/low: 28 months versus 15 months (*p* = 0.046)). Conversely, a study conducted by Shin and colleagues demonstrated that PD-L1 expression was independently associated with shorter survival in patients with metastatic RCC after VEGF-TKI treatment and significantly related to lack of VEGF-TKI responsiveness (*p* = 0.012) [127].

Overall, PD-L1 expression seems not to assume a predictive role when PD-1 or PD-L1 inhibitor are combined with TKIs [128,129]. A recent clinical trial assessed the efficacy of a combination strategy including atezolizumab, bevacizumab, carboplatin, and paclitaxel (ABCP) in metastatic non-squamous NSCLC patients. Notably, for patients without epidermal growth factor receptor (*EGFR*) or anaplastic lymphoma kinase (ALK) variations, ABCP group had prolonged recurrence-free survival (RFS) (HR = 0.77, *p* < 0.05, in PD-L1− patients) and OS (HR = 0.78, *p* = 0.02, in PD-L1− and PD-L1+ patients) regardless of PD-L1 status in comparison with BCP group. Due to enhanced migration of neo-antigen specific T cell and attenuated immune suppression caused by anti-angiogenesis and other treatments, it is difficult to predict alteration of immune microenvironment of PD-L1−patient post combination treatment [130]. In the context of combination of multiple drugs, the predictive value of PD-L1 expression is vague and deserves further investigation.

## 3. Combined Therapy in Clinical Practice: Where We Are Now

Based on biological evidences, many clinical trials evaluated immunotherapy and anti-angiogenics combined treatment in several subsets of tumors.

### 3.1. Renal Cell Carcinoma (RCC)

Both immunotherapy and anti-angiogenic agents have early emerged as a valid therapeutic strategy in the treatment of metastatic RCC (mRCC), if given separately.

Efforts have recently been made in order to determine potential synergistic effects of these two treatment modalities. As such, the open-label, parallel-cohort, multicenter phase I Checkmate 016 trial recently tested the combination of sunitinib or pazopanib plus nivolumab for advanced or mRCC with clear cell component, in both first and second line settings. In this study, combination treatment resulted in a major clinical benefit but with unacceptable toxicities, with 82% of patients experiencing G3–G4 adverse events (AEs) in the nivolumab + sunitinib arm (Table 1) [131]. 

Considering the toxicities reported, a still ongoing phase II trial is evaluating whether adding nivolumab as maintenance therapy would improve overall survival (OS), after disease control achieved with a first line TKI (either sunitinib or pazopanib) (Table 2).

Combining immunotherapy and anti-angiogenics also led to satisfying results. Two recent trials evaluated combined treatment with axitinib and immunotherapy (pembrolizumab or avelumab) compared with sunitinib in first-line setting advanced RCC. The multicenter, randomized, open-label, phase III JAVELIN Renal 101 trial compared axitinib (5 mg twice per day, orally, continuous dosing schedule) + avelumab (10 mg/kg e.v. every 2 weeks) versus sunitinib (50 mg orally, for 4 weeks on a 6-week cycle) as first line setting for advanced RCC with clear cell component (Table 1). Co-primary endpoints of the trial were median progression free survival (mPFS) and median OS (mOS) in PD-L1 positive subgroups. After a median follow-up of 11.6 months, experimental arm resulted in longer mPFS in both PD-L1 positive subgroup and in the overall population (mPFS in PD-L1 positive group: 13.8 versus 7.2 months, HR 0.61, *p* < 0.001; mPFS in the overall population: 13.8 versus 8.4 months, HR 0.69, *p* < 0.001), in each prognostic subgroup [129]. Combined treatment did not result in higher toxicities. Even though the data regarding OS in the PD-L1 positive subgroups are still immature, the FDA has recently approved axitinib + avelumab treatment as first line therapy for mRCC patients [129].

The contemporary published KEYNOTE-426 also evaluated the benefits of combining immunotherapy with anti-angiogenic drug in first line setting of mRCC (Table 1). This randomized, open-label, phase 3 trial directly compared pembrolizumab (200 mg, flat dose every 3 weeks, e.v.) + axitinib (dosage as above) versus sunitinib (dosage as above). Two co-primary end points of the study were PFS and OS in the intention-to-treat (ITT) population. After a median follow-up of 12.8 months, patients in the pembrolizumab-axitinib arm showed a longer mPFS compared to that observed in the sunitinib arm (mPFS in ITT: 15.1 versus 11.1 months, HR 0.69, *p* < 0.001). In addition, preliminary data from this trial also demonstrated a benefit in terms of OS for the experimental arm (12-month OS in ITT: 89.9% versus 78.3%, HR 0.53, *p* < 0.0001). These data were also confirmed in both PD-L1 positive and negative subgroups as well as across any risk category, without significant increase in toxicities [132]. Even if these two phase III trials differed in terms of stratification factors, evaluation of PD-L1 status and co-primary end points, they both demonstrated that combining immunotherapy and anti-angiogenicanti-angiogenics resulted in a prolonged PFS regardless PD-L1 status and prognostic subgroup, without increasing toxicity.

Other combination treatments have also been investigated. In the recently published multicenter, open-label phase III IMmotion 151 trial, it has been reported that combining bevacizumab and atezolizumab was superior to sunitinib in the first-line setting of advanced RCC (Table 1). In this trial, untreated metastatic renal cancer patients were randomized in a 1:1 ratio to either atezolizumab (1200 mg flat dose e.v. every 3 weeks) + bevacizumab (15 mg/kg, e.v. every 3 weeks) or sunitinib (dosage as above). Interestingly, both sarcomatoid as well as clear cell histology were included. Moreover, both PD-L1 positive (≥1%) and negative (<1%) patients were included Co-primary endpoints of the study were investigator-assessed PFS in PD-L1 positive disease and OS in ITT populations. After a median follow-up of 15 months, this trial reached only one of the two co-primary endpoints. In fact, a statistically meaningful advantage in terms of mPFS was obtained from the combined therapy, both in the PD-L1 positive subgroup and in the ITT population (mPFS in PD-L1 positive: 11.2 months in the atezolizumab plus bevacizumab group versus 7.7 months in the sunitinib group, HR 0.74, *p* = 0.0217; mPFS in ITT: 11.2 months in atezolizumab–bevacizumab vs. 8.4 months in sunitinib, HR 0.83, *p* = 0.02). Strikingly, patients with sarcomatoid histology benefited the most from combined therapy, independently from PD-L1 status (HR in PD-L1 positive patients 0.46, HR in ITT 0.56). However, OS in ITT and PD-L1 positive group did not reach statistical significance at the ad interim analysis. Even if more updated data on OS are still awaited, atezolizumab combined with bevacizumab is not yet approved for the treatment of mRCC at the moment this review was written [128].

Multiple ongoing trials are currently evaluating the role of combining cabozantinib with immunotherapy in first and second line settings of metastatic RCC. In this context, encouraging preliminary results were recently reported from a phase I/II trial assessing the activity of a combination therapy with pembrolizumab + cabozantinib in previously treated patients (Table 2) [133]. Moreover, promising preliminary data were also obtained from an ongoing phase I trial evaluating the efficacy of cabozantinib + nivolumab +/− ipilimumab in patients with metastatic urothelial carcinoma or RCC (Table 2) [134]. However, to date data are immature and results from further trials are awaited.

Taken together, these evidences suggest that combining immunotherapy and anti-angiogenic agents might represent a valid therapeutic strategy in metastatic RCC.

### 3.2. Non Small Cell Lung Cancer (NSCLC)

In the treatment strategy of NSCLC, both antiangiogenic agents and immune checkpoints inhibitors are routinely used. In fact, bevacizumab is currently approved for the first line treatment of locally advanced or metastatic non-squamous non oncogene addicted NSCLC, in combination with platinum-based chemotherapy and for patients with contraindications or ineligibility to immune checkpoint inhibitors treatment [135,136]. 

Non-oncogene-addicted NSCLC patients are also eligible for immunotherapy treatment. In this context, pembrolizumab and atezolizumab are currently approved in first as well as in subsequent lines of treatment, alone or combined with chemotherapy, while nivolumab finds indication in subsequent lines of treatment [118,137,138,139,140,141,142,143,144]. 

Relying on the overwhelming evidence on the efficacy of immunotherapy and anti-angiogenic agents when given separately, clinical trials evaluating the role of a combined strategy are currently being investigated also for NSCLC. In 2018, Socinski et al. demonstrated that adding atezolizumab to bevacizumab + carboplatin + paclitaxel as first-line therapy resulted in an augmented progression-free survival (PFS) and overall survival (OS) for patients with non-squamous histology advanced NSCLC (Table 1) [130]. In the open-label, multicentric, phase III IMpower 150 trial patients with chemotherapy-naive metastatic, non-squamous NSCLC were randomized in 1:1:1 ratio to one of the following induction regimens: ACP (Atezolizumab 1200 mg/mq, Carboplatin AUC 6, Paclitaxel 200 mg/mq, e.v. every 3 weeks), ABCP (Atezolizumab, carboplatin, paclitaxel with the previously described doses + Bevacizumab 15 mg/kg e.v. every 3 weeks), or BCP (bevacizumab-carboplatin-paclitaxel with previously described doses, e.v. every 3 weeks). Induction phase lasted from four to six cycles and was followed to a maintenance phase, in which atezolizumab and/or bevacizumab were administered until progression of disease or unacceptable toxicity. Enrolled patients could have any PD-L1 status Patients with *EGFR* mutations (exon 19 deletion or L858R mutation) as well as ALK translocation have been included in the trial after progression to at least one prior TKI therapy. Co-primary endpoints were PFS, both in ITT wild-type (WT) population (ITT-WT population: without *EGFR* mutations or ALK translocation) and among patients in the WT population who had high expression of an effector T-cell (Teff) gene signature in the tumor (Teff-high WT population), as well as OS in ITT-WT population. The Teff gene signature was defined as the expression of PD-L1, CXCL9, and IFN-γ messenger RNA. To date, only results obtained from comparison between ABCP and BCP are available. After a median follow-up of 15.4 months, people in ABCP arm experienced a higher PFS, both ITT-WT population and Teff-high WT population (mPFS in ITT-WT population: 8.3 versus 6.8 months, HR 0.62, *p* < 0.001; mPFS in Teff-high WT population: 11.3 versus 6.8 months, HR 0.51, *p* < 0.001). The same benefit was observed for the co-primary end point: mOS in ITT-WT population was 19.2 months versus 14.7 months for ABCP versus BCP, respectively (HR 0.78, *p* = 0.02). Interestingly, adding atezolizumab to BCP regimen resulted in an advantage in terms of PFS across all subgroups, including those with *EGFR* or *ALK* genetic alteration, among patients with low or high PD-L1 expression, those with low Teff gene-signature expression, and those with liver metastases [130]. For the first time, this trial has indisputably demonstrated a benefit of combining immunotherapy, anti-angiogenics and chemotherapy. As such, ABCP gained the FDA and european medicines agency (EMA) approval as first line treatment for metastatic non-squamous NSCLC, regardless PD-L1 expression. ABCP is also approved for *EGFR* mutant or ALK translocated patients, after failure to appropriate targeted therapies.

Other strategies of combining immunotherapy and anti-angiogenics are currently being investigated.

The randomized, open label, phase I/II KEYNOTE-021 trial is currently investigating the safety and efficacy of combining pembrolizumab with one of the following treatment: carboplatin-paclitaxel, carboplatin-paclitaxel + bevacizumab, carboplatin-pemetrexed, ipilimumab or *EGFR* TKI gefitinib/erlotinib. Patients in this trial must have locally advanced (stage IIIB) or metastatic NSCLC with no prior systemic treatment. To date, however, efficacy of pembrolizumab-carboplatin-pemetrexed are the only available results [145]. Data on combining pembrolizumab to bevacizumab are still awaited.

A still recruiting open-label phase II study is aiming at demonstrate activity and safety of pembrolizumab with bevacizumab for treating patients with brain metastasis (both from melanoma and from NSCLC). Results will enlighten treatment options for this category of patients (Table 2). Nintedanib is a small molecule that inhibits, among others, VEGFR1-3. This drug is currently approved as second line treatment of locally advanced or metastatic NSCLC, in association with docetaxel and for adenocarcinoma histotype [146]. An ongoing non randomized phase I/II clinical trial is currently evaluating the combination of nintedanib with nivolumab-ipilimumab for advanced or metastatic NSCLC (Table 2).

Taken together, these evidences provide a strong rationale of using combination treatments in NSCLC patients.

### 3.3. Colorectal Cancer (CRC)

Several anti-angiogenic agents are currently approved for the treatment of metastatic CRC. In this context, bevacizumab finds indication both in first line and in second line settings, combined with fluoroypirimidines-based chemotherapy [40,45,147,148,149]. Moreover, bevacizumab has recently been investigated as maintenance therapy after first line not progressing disease, with contrasting results [150,151]. More recently, aflibercept, ramucirumab, and regorafenib found indication in the treatment of metastatic CRC [152,153,154,155,156,157].

Immunotherapy has recently prompted as potential treatment option for metastatic CRC patients harboring deficit in mismatch repair genes (MMR-deficient, dMMR). Basing on results of multiple phase II trial, the FDA recently approved nivolumab and pembrolizumab in patients with deficiency in MMR genes; however, the EMA approval for these cases is still under evaluation [158,159,160].

Currently, multiple clinical trials are investigating the effectiveness of combining standard therapy, including anti-angiogenic agents, to immunotherapy. Despite this, few data on these trials are yet available. A still ongoing phase I trial is currently evaluating the potential role of combining atezolizumab with bevacizumab and investigator’s choice chemotherapy for metastatic colorectal patients with MSH-I, with encouraging preliminary results (Table 2) [161]. Moreover, the phase II MODUL trial is currently investigating the benefits of adding atezolizumab to bevacizumab and fluropyrimidines in first line colorectal patients, regardless the mismatch repair gene status.

Despite the small number of patients, these results led to the phase II MODUL trial. In this trial, patients with untreated metastatic CRC received a prior induction chemotherapy with FOLFOX-bevacizumab for either eight cycles or six cycles. In the latter case, two more cycles of 5-FU/LV where subsequently added. Patients achieving at least a stable disease were then enrolled in specific cohorts of the trial depending on their molecular mutational status. d-MMR/MSH-I was not a mandatory inclusion criteria. In 2018, results from the BRAF-wild type cohort of patients were presented at the European Society for Medical Oncology (ESMO) conference. This cohort enrolled 445 patients who were randomized in a 2:1 ratio to experimental arm (5-FU/LV + bevacizumab + atezolizumab) or the standard of care (5-FU/LV + bevacizumab). Interestingly, almost 98% of patients in each arm displayed MSS. In this trial, the addition of atezolizumab to bevacizumab + 5-FU/LV did not result in differences in terms of PFS and OS (mPFS for 5-FU/LV+ atezolizumab + bevacizumab versus 5-FU/LV+ bevacizumab: 7.20 versus 7.39 months, HR 0.96, *p* = 0.727, mOS for 5-FP/LV+ atezolizumab + bevacizumab versus 5-FP/LV+ bevacizumab: 22.05 versus 21.91 months, HR 0.86, *p* = 0.283) (Table 1) [162].

However, the ongoing phase III Colorectal Cancer Metastatic dMMR Immuno-Therapy (COMMIT) trial is also evaluating the efficacy of atezolizumab + bevacizumab in the first line setting. In this trial, people with untreated metastatic CRC and d-MMR are randomized to receive mFOLFOX6 + atezolizumab + bevacizumab, mFOLFOX6 + bevacizumab or atezolizumab alone. Results from this trial will definitively highlight the role of immunotherapy and anti-angiogenics as upfront therapy for colorectal cancer patients with d-MMR (Table 2) [163].

Atezolizumab+ bevacizumab is also under evaluation in subsequent lines of therapy, as well as for neoadjuvant treatment for rectal cancer patients.

Given the lack of well-proven data, no conclusion could be made on the real effectiveness of combined treatment in colorectal cancer patients.

### 3.4. Gastrointestinal Malignancies

In advanced gastric or gastroesophageal adenocarcinomas, ramucirumab is approved as a second line of therapy, either alone or combined with paclitaxel [44,164].

Recently, the FDA also approved pembrolizumab in subsequent lines of therapy, both as third line therapy in case of highly PD-L1 expressing tumors, and for second line therapy for d-MMRI patients with no other treatment options. However, these approval followed two phase II trials, and pembrolizumab has not yet been approved by EMA [160,165].

Preliminary results of a still ongoing phase I study combining pembrolizumab with ramucirumab are available. This multi-cohort trial enrolled 28 treatment-naive patients with gastric or gastroesophageal junction adenocarcinoma to receive ramucirumab (8 mg/kg on days 1 and 8 e.v. every 3 weeks) + pembrolizumab (200 mg on day 1 e.v. every 3 weeks). Although the large majority of patients were PD-L1 positive, high PD-L1 expression was not a mandatory inclusion criteria. This trial aimed at demonstrating the safety and tolerability of the study treatment, but efficacy analysis were secondary end points. After a median duration of treatment of 4.3 months, and a median follow up of 8.1 months, these preliminary results showed disease control rate of 68%, a mPFS of 5.3 months and a median duration of response of 10 months. In terms of toxicities, 96% of patients experienced adverse events, and 61% of patients had a grade 3 related toxicity. However, mOS has not yet been reached, and further data of this still ongoing trials are awaited [166] (Table 2).

A not yet recruiting open label, phase II SEQUEL trial will aim at demonstrate the effectiveness of experimental treatment with pembrolizumab-ramucirumab-paclitaxel in patients with advanced gastric or gastroesophageal adenocarcinoma after progression on at least one prior line of therapy for metastatic disease, independently from PD-L1 status.

Given the lack of conclusive data, no conclusion could be made on the efficacy of combined treatment in gastric or gastroesophageal junction adenocarcinoma. (Table 2).

### 3.5. Melanoma

Immunotherapy has widely spread as a treatment option for cutaneous melanoma long before the approval of immune checkpoint inhibitors. IFN-alpha was among the first systemic therapy to be approved after resection of melanoma in adjuvant setting, although with the recent approval of anti- PD-1/PD L1, this treatment lost its appeal. In fact, pembrolizumab and nivolumab recently gained the FDA as well as the EMA approval as adjuvant treatment for node-positive melanoma after complete resection of disease. Ipilimumab is also FDA approved as adjuvant treatment of this disease, although this indication has not been followed by EMA approval due to a higher toxicity profile of anti-CTLA4 compared to anti-PD-1/PD-L1. Moreover, ipilimumab, nivolumab, ipilimumab+nivolumab and pembrolizumab are currently approved for the treatment of advanced unresectable melanoma [167]. Even if immune checkpoint inhibitors sauntered onto the scene, other strategies for stimulating immune system are currently being investigated for the treatment of this disease. As such, both IL-2 and bacille Calmette–Guérin (BCG) have been investigated for the treatment of in-transit melanoma disease, when surgery is not feasible. However, systemic effects of locally injected IL-2 and BCG have been observed so that this strategy is still to be considered as experimental. Patients with unresectable, in-transit melanoma disease have also been treated with intralesional or perilesional granulocyte macrophage-colony stimulating factor (GM-CSF), with objective results in some small clinical studies. This led to the development of talimogene laherparepvec (T-VEC), a modified herpes virus 1 (HSV-1) that specifically enter, replicate and synthesize GM-CSF into tumor cells. T-VEC demonstrated its efficacy in advanced unresectable melanoma, thus gaining the FDA and EMA approval for the treatment of stage IIIB-IIIC-IV unresectable melanoma [168].

Although bevacizumab is not approved for the treatment of cutaneous melanoma, multiple data suggests its efficacy also in this disease [169,170,171]. Moreover, a previously reported phase II trial demonstrated a clinical benefit of bevacizumab combined with IFN-alpha [172]. As such, a recently published phase I trial aimed at evaluating the safety and efficacy of combining the novel immune checkpoint ipilimumab with bevacizumab for unresectable stage III or IV melanoma patients (Table 1). This trial enrolled 46 patients; most of them were males with ECOG PS 0, metastatic disease with a median number of site of disease of 3 organs. Prior treatments were allowed. Enrolled patients were then divided into 4 cohorts depending on the doses of ipilimumab and bevacizumab received. Primary end points were safety and tolerability, however efficacy data are available. At a median follow-up of 17.3 months, this trial demonstrated a best ORR of 19.6%. Disease control rate was 67.4% whereas time to progression was 9 months with a median OS of 25.1 months. Interestingly, this trial also demonstrated a change in tumor microenvironment after exposure to combined therapy. In fact, treatment with ipilimumab plus bevacizumab resulted in morphologic changes in intratumoral endothelia with higher E-cadherin expression as well as rounded and columnar CD31+ cells compared with pretreatment or post-treatment samples from patients receiving ipilimumab alone. Higher CD31 expression was also observed at the interendothelial junctions. Moreover, these endothelial changes were associated with extensive immune cell infiltration of tumors, which translated into a higher concentration of CD8+ T cells and CD163+ dendritic macrophages in patients receiving both treatment compared to biopsies obtained from those receiving ipilimumab alone. Finally, changes in terms of immune cells populations were also seen in experimental blood of patients receiving combined therapy, with a higher rate of memory immune cells. As such, this trial firstly demonstrated in a translational way a clinical benefit of combining bevacizumab to ipilimumab in advanced melanoma [173]. However, results from a still ongoing phase II trial comparing ipilimumab with ipilimumab + bevacizumab in this subset of patients are awaited (Table 2). Finally, still recruiting studies are evaluating the role of combining atezolizumab/pembrolizumab with bevacizumab for metastatic melanoma patients.

These preliminary results highlight a potential synergistic effect of immune checkpoints inhibitors and antiagiogenics in the treatment of advanced melanoma. However, further studies are awaited.

### 3.6. Breast Cancer

Bevacizumab is also approved as first line treatment of HER-2 negative metastatic breast cancer, combined with either paclitaxel or capecitabine. Vaccine-based immunotherapy has largely been an unsuccessful therapeutic option for breast cancer patients [174]. More recently, clinical results were obtained with immune checkpoint inhibitors, especially for triple negative breast cancer. In fact, atezolizumab combined with paclitaxel gain the FDA and EMA approval as first line therapy for metastatic triple negative breast cancer patients harboring high PD-L1 expression [175]. Early results obtained from the phase III KEYNOTE-522 trial also demonstrated a clinical advantage from the usage of pembrolizumab as neoadjuvant agent in the treatment of locally advanced triple negative breast cancer [176]. Immunotherapy has also been investigated for patients progressing to a first line therapy containing bevacizumab, in order to determine whether immune checkpoint inhibitors could restore sensitivity to anti-angiogenics. A recently published phase Ib study enrolled patients with metastatic HER2 negative breast cancer who experienced progression after at least 6 weeks of a bevacizumab-containing first line treatment. The anti-PD-L1 durvalumab (10 mg/kg e.v. every 2 weeks) was added to bevacizumab (10 mg/Kg e.v. every 2 weeks) in these patients and peripheral-blood mononuclear cells (PBMCs) were phenotyped in order to monitor 24 lymphoid and non-neutrophil myeloid subpopulations before the first dose of durvalumab and every 4 weeks until PD. Primary end point of the study was PFS, secondary end points were toxicity and relative changes in PBMCs subpopulations. Adding durvalumab resulted in mPFS of 133 days, with no new toxicity observed. Interestingly, patients who maintained stable disease after the first evaluation (at 2 months) showed from 1.2- to 3.5-fold increase in CD8 effector memory T-cells (CD8EM) in PBMCs compared to what observed in baseline. At the contrary, this change in peripheral blood was not observed in those who experienced progression of disease (Table 1) [177]. Nevertheless, little evidence of a proved efficacy for this setting are yet available and further studies demonstrating major clinical benefit of immunotherapy and anti-angiogenics are awaited.

### 3.7. Urothelial Carcinoma

Urothelial carcinoma was one among the firsts that experimented the usage of vaccines. Still, BCG vaccine finds indication as adjuvant treatment for some cases of non-muscle-invasive urothelial carcinoma, both as induction and as maintenance therapy. Moreover, pembrolizumab and atezolizumab are currently FDA approved for the first-line treatment of the locally advanced and metastatic urothelial carcinoma, either for those cisplatinum-unfit with high expression of PD-L1 or for any platinum-unfit patients regardless from the PD-L1 status. Finally, pembrolizumab, atezolizumab, nivolumab, durvalumab, and avelumab are FDA approved as subsequent lines of therapy for platinum-progressing patients [178]. Although high levels of VEGF correlate with a worse prognosis, no anti-angiogenic drug is currently approved in this type of solid tumor [179]. Bevacizumab has been tested for both neoadjuvant and advanced disease, without any clear clinical benefit [180,181]. The same unconvincing results were obtained with other anti-angiogenics such as sunitinib, pazopanib, vandetanib, or cabozantinib [182,183,184]. Ramucirumab was the only anti-angiogenic drug with a demonstrated clinical benefit for metastatic urothelial carcinoma patients, combined with docetaxel as second line treatment [185].

Novel anti-VEGFR TKIs are currently being investigated for urothelial carcinoma. Preliminary results of a still ongoing phase Ib/II trial demonstrated that combining lenvatinib (a VEGFR1-2-3 TKI inhibitor) with pembrolizumab resulted in high ORR and PFS. In this multicentric, open label study, patients with metastatic urothelial carcinoma received lenvatinib + pembrolizumab irrespectively from their PD-L1 status as well as prior systemic treatments. Prior treatments were allowed and PD-L1 positive status was not mandatory. (Table 2). An ongoing phase 3 trial is directly evaluating the efficacy of pembrolizumab + lenvatinib versus pembrolizumab + placebo as first line treatment for locally advanced or metastatic urothelial carcinoma. This trial will enroll both cisplatin ineligible patients with tumors highly expressing PD-L1, and patients ineligible to any other platinum agents regardless the PD-L1 expression (Table 2).

Given the lack of evidence, no conclusion should be made whether adding anti-angiogenics to immunotherapy could results in major clinical benefits.

## 4. Conclusions

Recently, an increasing amount of preclinical data indicates that vessel normalization strategies can improve the aberrant structure and function of tumor blood vessels and can also result in reduction of tumor hypoxia, reduction of function of suppressive cells, and promotion of antitumor activity of immune effector cells.

Many studies are focusing on reprogramming tumor microenvironment to become more immune-stimulatory by combining anti-angiogenic drugs and immune check point inhibitors. In this contest, anti-angiogenic drugs seem to block the negative immune signals by increasing ratio of anti-/pro-tumor immune cells and decreasing immune checkpoints expression, while immunotherapy seems to restore immune-supportive microenvironment and to promote vascular normalization increasing lymphocyte infiltration and activation.

Moreover, evidence from studies that observed an increasing PD-L1 expression in anti-angiogenetic treated tumors suggest that immune system could be involved and could promote resistance to anti-angiogenic agents. Therefore, strategies targeting the immunosuppressive PD-1/PDL-1 signaling in anti-angiogenesis resistant tumors are emerging.

With the increased understanding of tumor escape mechanisms, predictive biomarkers such as PD-L1 expression have to be investigate in order to can use them for patient selection and precision therapy.

Currently, numerous clinical studies are under way to test the impact of simultaneous inhibition of angiogenesis and immune checkpoints. The preliminary results are promising and initial phase I/II data reported the superiority of combined treatment compared to monotherapy. However, final outcomes of large phase III studies remain awaited, before final conclusions can be drawn.

The best results from clinical trials on combined therapy regard RCC and NSCLC but in the next years we expect to obtain more consistent results also from ongoing studies about the other malignancies.

## Figures and Tables

**Table 1:** Clinical trials combining immunotherapy and antiagiogenics.

Authors, Year	Disease/Setting	Clinical Trial/Phase	Study Design	Total No. of Patients, mFU	Primary End Points	Results
Brian I. Rini et al. The Lancet 2019 [128]	LA or mRCC (clear cell, sarcomatoid), first line	IMmotion 151, phase III, NCT02420821	R (1:1), OL, atezolizumab + bevacizumab vs. sunitinib	951 pts, mFU: 15 m	IA PFS in PD-L1+ pts and OS in ITT population	IA mPFS in PD-L1+: 11.2 vs. 7.7 months (HR 0.74, *p* = 0.0217) mOS in ITT: 33.6 vs. 34.9 (HR 0.93, *p* = 0.4751)
Motzer et al. NEJM 2019 [129]	mRCC (clear cell), first line	Javeline Renal 101, phase III, NCT02684006	R (1:1), OL, avelumab + axitinib vs. sunitinib	866 pts, mFU: 11.6 m and 10.7 m	PFS and OS in PD-L1 + pts	mPFS in PD-L1+: 13.8 vs. 7.2 months (HR 0.61, *p* < 0.001) mOS in PD-L1+: data immature
Mark A. Socinski et al. NEJM 2018 [130]	Non-squamous NSCLC, First line	IMpower 150, phase III, NCT02366143	R (1:1:1), OL, ACP vs. BCP vs. ABCP x4-6 cycles (induction), followed by A or B or A+B (maintenance)	1202 pts, mFU: 15.4 months (ABCP) and 15.5 months (BCP)	IA PFS (both in ITT-WT and in Teff- high WT populations) and OS in ITT- WT population	mPFS in ITT-WT: 8.3 vs. 6.8 months (HR 0.62, *p* < 0.001); mPFS in Teff-high WT: 11.3 vs. 6.8 months (HR 0.51, *p* < 0.001); mOS in ITT-WT: 19.2 vs. 14.7 months (HR 0.78, *p* = 0.02)
Amin A. et al. J Immunother Cancer 2018 [131]	mRCC (clear cell or non-clear cell), first and subsequent lines	Checkmate 016, phase I, NCT01472081	OL, nivolumab + sunitib (N+S), nivolumab + pazopanib (N+P)	N+S: 33 pts mFU: 50 m N+P: 20 pts mFU: 27.1 m (closed early)	Safety and tolerability	AEs in N+S: 100% G3-4 AEs in N+S: 82% AEs N+P: 100% G3-4 AEs in N+P: 70%
Brian I. Rini et al. NEJM 2019 [132]	mRCC (clear cell), first line	KEYNOTE-426, phase III, NCT02853331	R (1:1), OL, pembrolizumab + axitinib vs. sunitinib	861 pts, mFU:12.8m	PFS and OS in ITT population	mPFS in ITT: 15.1 vs. 11.1 months (HR 0.69, *p* < 0.001) mOS in ITT: data immature
						
A.Grothey et al. Ann of Oncol. 2018 [156]	mCRC, first line, Results from Cohort 2 available (BRAFwt)	MODUL, Phase II, NCT02291289	Umbrella, FOLFOX+ B x 16 weeks (induction), then R (2:1) to FP/B vs. FP/B +A (maintenance in Cohort 2).	696 pts (445 pts in cohort 2) mFU Cohort 2: 10.5 months	PFS per investigator	mPFS: 7.39 vs. 7.20 months (HR 0.96, *p* = 0.727)
F. S. Hodi et al. Cancer Immunol Res. 2014 [173]	Metastatic melanoma, First and subsequent lines	Phase I, NCT00790010	OL, non-R, 4 cohorts, Ipilimumab (induction and maintenance), bevacizumab (continuous)	46 pts, mFU: 17.3 months	Primary end points: Safety and tolerability, Secondary end points: BORR, DCR, TTP, mOS	MTD: cohort 2 (ipilimumab 10 mg/kg + bevacizumab 15 mg/kg) Overall G3-4: 28.3% BORR: 19.6% DCR: 67.4% TTP: 9 months mOS: 25.1 months
M. Quintela-Fandino et al. JCO 2018 [177]	mBC, subsequent lines	Phase Ib, NCT02802098	OL, single arm, durvalumab + bevacizumab after PD to taxane+ bevacizumab regimen	24 pts mFU: NA	PFS	mPFS: 76 days

R = randomized, OL = Open Label, mPFS = median Progression Free Survival, mOS = median Overall Survival, mFU = median Follow Up, pts = patients, IA = investigator assessed, LA = locally advanced, m = months, A = atezolizumab, B = bevacizumab, ABCP = atezolizumab + bevacizumab + carboplatin + paclitaxel, BCP = bevacizumab + carboplatin + paclitaxel, ACP = atezolizumab + carboplatin + paclitaxel, ITT = intention to treat, WT = wild type, ET = experimental treatment, FP = fluoropyrimidine, G = gastric, GEJ = gastroesophageal junction, MTD = maximum tolerated dose, BORR = best overall response rate, DCR = disease control rate, TTP = time to progression, BC = breast cancer, NA = not available.

**Table 2:** Ongoing trials.

Combination Drugs	Disease Condition	Study Phase	Status at Time of Search	Clinical Trial ID
Nivolumab as maintenance therapy after sunitinib/pazopanib	LA/mRCC Maintenance therapy	Phase II	Active, not recruiting	NCT02959554
Cabozantinib + nivolumab +/− ipilimumab	LA/m urothelial carcinoma, mRCC, other genitourinary tumors subsequent lines	Phase I	recruiting	NCT02496208
Pembrolizumab + cabozantinib	mRCC subsequent lines	Phase I/II	recruiting	NCT03149822
Pembrolizumab + CT ^1^ + bevacizumab/ipilimumab/antiEGFR	LA/m NSCLC First line	Phase I/II	Active, not recruiting	NCT02039674
Pembrolizumab + bevacizumab	untreated brain metastases from melanoma or NSCLC	Phase II	recruiting	NCT02681549
Nintedanib + ipilimumab + nivolumab	mNSCLC First and subsequent lines	Phase I/II	recruiting	NCT03377023
Atezolizumab + bevacizumab +/− CT ^2^ Atezolizumab + CT ^3^	Advanced solid tumors Subsequent lines	Phase I	Active, not recruiting	NCT01633970
Atezolizumab + bevacizumab + mFOLFOX6	mCRC first line	Phase III	recruiting	NCT02997228
Ramucirumab + Pembrolizumab	LA/m G or GEJ adenocarcinoma NSCLC BTC Urothelial carcinoma First and Subsequent lines	Phase I	Active, not recruiting	NCT02443324
Ramucirumab + pembrolizumab + paclitaxel	LA/m G or GEJ adenocarcinoma Subsequent lines	Phase II	Active Not yet recruiting	NCT04069273
Ipilimumab + bevacizumab	LA/m melanoma First and subsequent lines	Phase II	Active, not recruiting	NCT01950390
Lenvatinib + pembrolizumab	Advanced solid tumors Subsequent lines	Phase I/II	Active, not recruiting	NCT02501096
Lenvatinib + pembrolizumab	LA/m urothelial carcinoma First line	Phase III	recruiting	NCT03898180

LA = locally advanced, m = metastatic, RCC = renal cell carcinoma, G = gastric, GEJ = gastroesophageal junction, BTC = biliary tract adenocarcinoma, ^1^ CT = CBDCA + paclitaxel +/− bevacizumab, CBDCA + pemetrexed; ^2^ CT = FOLFOX; ^3^ CT = Carboplatin + Paclitaxel, Carboplatin + Pemetrexed, Carboplatin + Nab-paclitaxel, Nab-paclitaxel.
